# Global Trends and Insights Into Opioid Utilization for Postoperative Pain Management: A Bibliometric Analysis (2014–2024)

**DOI:** 10.1155/prm/1141767

**Published:** 2025-09-24

**Authors:** Lingxian Kong, Jiangang Hu

**Affiliations:** Department of Anesthesiology, Tianshui First People's Hospital, Tianshui 741000, China

**Keywords:** bibliometric analysis, opioids, postoperative analgesia, research trends

## Abstract

**Background:** Opioids are a class of potent analgesics extensively utilized for the management of moderate to severe pain. They are integral to postoperative analgesia, effectively mitigating pain following surgical interventions. The present study aims to undertake a comprehensive bibliometric analysis to evaluate research trends and focal areas within the domain of opioid use and postoperative analgesia.

**Methods:** A bibliometric analysis was conducted using the Web of Science Core Collection to gather literature from 2014 to 2024. Analysis of publication trends, research hotspots, and collaboration networks was conducted using VOSviewer, CiteSpace, and the R package “bibliometrix.”

**Results:** The search yielded 5383 relevant articles, indicating a consistent upward trend in research volume, with a significant increase commencing in 2019. The USA emerged as the leading contributor, with Harvard University identified as the foremost institution. The journal Anesthesia and Analgesia was recognized as the most prominent publication in this field, while the influential author was Meissner Winfried. Analyses of keyword identified four clusters, such as complications management, nonopioid analgesics, clinical validation of opioid-free anesthesia (OFA) and pharmacokinetics of opioids. Keywords burst analysis showed emerging interests in “enhanced recovery after surgery,” “prescription,” and “erector spinae plane block.”

**Conclusion:** This bibliometric analysis mapped the scientific landscape of opioids in postoperative analgesia. The research hotspots included importance of complications management, nonopioid analgesics, clinical validation of OFA, and pharmacokinetics of opioids. Future studies should improve patient outcomes through individual-based multimodal analgesia with more efficacy and safety.

## 1. Introduction

Postoperative pain is a common complication experienced by surgical patients, influenced by various factors such as the type of surgery, extent of trauma, age, gender, psychological state, and individual pain sensitivity [1]. Inadequate pain control can lead to numerous adverse outcomes, including the development of chronic pain, impaired immune function, delayed recovery, and prolonged hospital stays, which in severe cases may increase the risk of postoperative complications, significantly impacting patients' quality of life and healthcare costs [2]. In recent years, the use of opioids in pain management strategies has been the focus of extensive research and debate. Opioids remain among the most commonly used options for managing moderate to severe postoperative pain [3].

Despite the effectiveness of opioids in relieving postoperative pain, their potential side effects and risk of dependency pose significant challenges. Adverse effects such as nausea, vomiting, respiratory depression, and long-term addiction risk limit their clinical utility [4]. To address these issues, research has increasingly focused on developing novel opioid analgesics that minimize side effects while enhancing efficacy. For example, biased *μ*-opioid receptor agonists like oliceridine and tapentadol have been approved, demonstrating fewer side effects [5]. Additionally, cebranopadol, a multitarget analgesic, and NFEPP, a peripherally acting opioid receptor agonist, have shown promising analgesic effects and safety profiles in preclinical studies [6]. To comprehensively understand the research trends and hotspots of opioids in postoperative analgesia, conducting a bibliometric analysis is valuable for uncovering the current landscape and future directions in this field.

Bibliometric analysis quantitatively evaluates academic literature, employing statistical and visualization techniques to reveal trends, hotspots, and collaboration networks. This approach enables researchers to identify key publications, grasp research dynamics, and establish a foundation for future investigations [7, 8]. Despite several bibliometric analyses have investigated postoperative pain management, such as various analgesia strategies [9, 10] and opioid-free anesthesia (OFA) [11, 12], none have specifically focused on opioid use for postoperative pain. There is a notable gap in bibliometric analyses specifically focused on opioids for postoperative analgesia. Our study aimed to explore research hotspots and trends in the field of opioids for postoperative analgesia over the past decade.

## 2. Materials and Methods

### 2.1. Search Strategies and Data Collection

A literature search on opioids in the field of postoperative analgesia was conducted using the Web of Science Core Collection (WoSCC), a comprehensive and authoritative database that indexes high-quality research across various disciplines, for publications from 2014 to 2024. The search strategy was as follows: (TS = (Opioids OR opiates OR opium OR buprenorphine OR butorphanol OR codeine OR diphenoxylate OR fentanyl OR heroin OR levorphanol OR methadone OR loperamide OR meperidine OR morphine OR naloxone OR nalbuphine OR nalmefene OR naltrexone OR oxycodone OR propoxyphene OR pentazocine OR tramadol OR tapentadol)) AND TS = (“postoperative pain” OR “postsurgical pain”) [13, 14]. To prevent inconsistencies from database updates, the literature retrieval was performed on October 17, 2024. The inclusion criteria were as follows: (1) original research articles written in English, (2) studies focusing on the integration of opioid utilization for postoperative pain management, and (3) publications with complete bibliographic data. Bibliographic information was exported in “rull record and cited references” and “plain text” formats, capturing publication and citation metrics, author details, institutional affiliations, geographic data, keywords, and journal characteristics. The literature search strategy was independently conducted by two reviewers. They then discussed and refined the search results together to develop the final search strategy. Subsequently, the reviewers screened the studies based on predefined inclusion criteria. Any discrepancies between the reviewers were resolved through discussion. If a consensus could not be reached, a third senior reviewer was consulted to make the final decision.

### 2.2. Statistical Analysis

Microsoft Excel 2019, the R package “bibliometrix” (version 3.2.1, https://www.bibliometrix.org), VOSviewer 1.6.20 (version 1.6.20), and CiteSpace (version 6.1. R2) were utilized for data analysis and visualization. Microsoft Excel was used to identify and calculate key bibliometric indicators, including annual publication quantity, citation frequency, average citation frequency, journal names with impact factors, publishing regions and institutions, and author contributions. The analysis of annual publication numbers was also conducted using Microsoft Excel. R package “bibliometrix” facilitated the visualization of publication outputs from corresponding authors across different countries and the article count from the top 10 institutions. VOSviewer was employed to visualize collaboration among countries, institutions, and authors, as well as co-occurrence and coupling networks of journals and keyword co-occurrence networks. CiteSpace was used to visualize the citation bursts of keywords in this study. The parameters were set as follows: time slicing from January 2014 to October 2024, with a slice length of one year. The node type selected was “keywords,” with a threshold of the top 5 keywords per slice. For pruning, the pathfinder algorithm and merged network pruning were applied. This visualization generated a keyword timeline map, highlighting research trends and bursts in the field of “opioids in postoperative analgesia.”

Several parameters from the WoSCC, including the h-index and g-index were employed to quantify the academic impact of individuals and journals [15, 16]. The h-index is a vital indicator for evaluating researchers' academic contributions and predicting their future scientific achievements. The g-index enhances this evaluation by giving more weight to highly cited articles, providing a better assessment of a researcher's impact. We also assessed journals using Impact Factor (IF) and Journal Citation Reports (JCR) in 2023.

## 3. Results

### 3.1. An Overview of Publications

**Figure 1:**
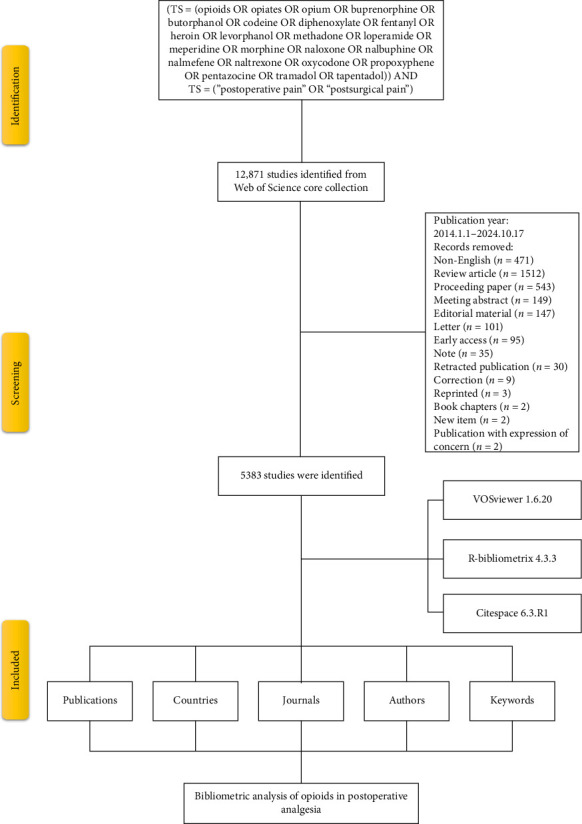
Flowchart of the literature screening process.

**Figure 2:**
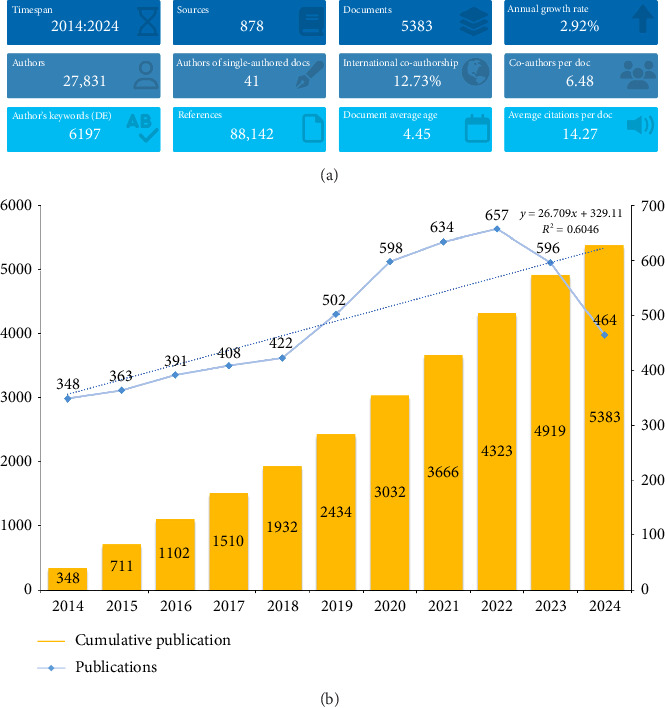
(a) The overall trend of relative publications. (b) Annual number of publications.

The study ultimately encompassed a total of 5383 original research articles published globally between 2014 and 2024, focusing on the topic of opioids in postoperative analgesia (Figure 1). These articles were authored by 27,831 contributors, published across 878 sources, and cited 88,142 references. The average annual growth rate of publications was 2.92%, with each author contributing an average of 6.48 articles. The rate of international collaboration reached 12.73%, and each article had an average age of 4.45 years, with a mean citation count of 14.27 per publication (Figure 2(a)).

The annual growth rate demonstrated a steady increase in research on opioids for postoperative analgesia. From January 2014 to August 2024, the number of published papers showed exponential growth. This trend was divided into two phases: Phase One, before 2018, with relatively slow progress and fewer than 500 annual publications on average, and Phase Two, starting in 2019, marked by a notable increase. Although the growth rate slightly slowed over the past two years, annual publications consistently exceeded 500. Remarkably, even with 2024 not yet complete, 464 papers had already been published, reflecting continued strong interest in the field (Figure 2(b)).

### 3.2. Global Collaboration Network of Countries

**Figure 3:**
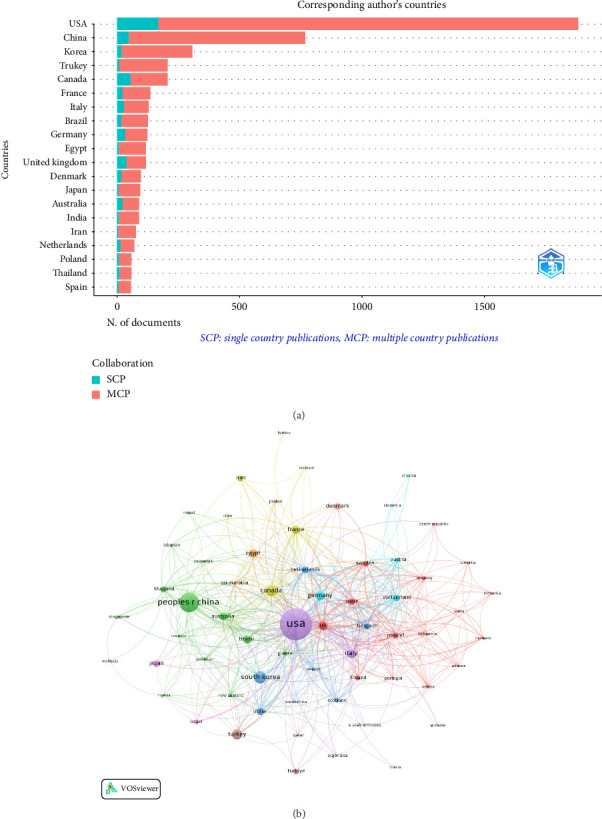
(a) Distribution of corresponding author's publications by country. The number of publications attributed to corresponding authors from different countries, distinguishing between single-country publications (SCP) and multiple-country publications (MCP). (b) Visualization map depicting the collaboration among different countries. The collaborative relationships between countries, with nodes representing countries, the size of nodes indicating publication count, and the thickness of links showing the strength of co-authorship collaborations.

The top 20 countries by publication volume, primarily in Asia, North America, and Europe, are shown in Figure 3(a) and Table 1. The USA led with 1884 publications (35.0%) and total citations of 31,954, followed by China with 768 publications (14.3%) and total citations of 7062 and South Korea with 308 publications (5.7%) and total citations of 3606. The USA and China accounted for almost half of the total research output in the field (Table 1). Publications were classified as single country publications (SCP) or multiple country publications (MCP). The MCP rates for the USA and China were 9% and 6.4%, respectively (Figure 3(a) and Table 1). A collaboration network was constructed among the 62 countries engaged in international research partnerships, each with a minimum of five articles. The USA led with 507 collaborations, followed by the UK with 213 and Canada with 168. Strong collaborative networks were also evident in the Netherlands, Denmark, Switzerland, and among Asian countries, including China, South Korea, and Japan (Figure 3(b)).

### 3.3. Publications and Collaborations Analysis of Institutions

**Figure 4:**
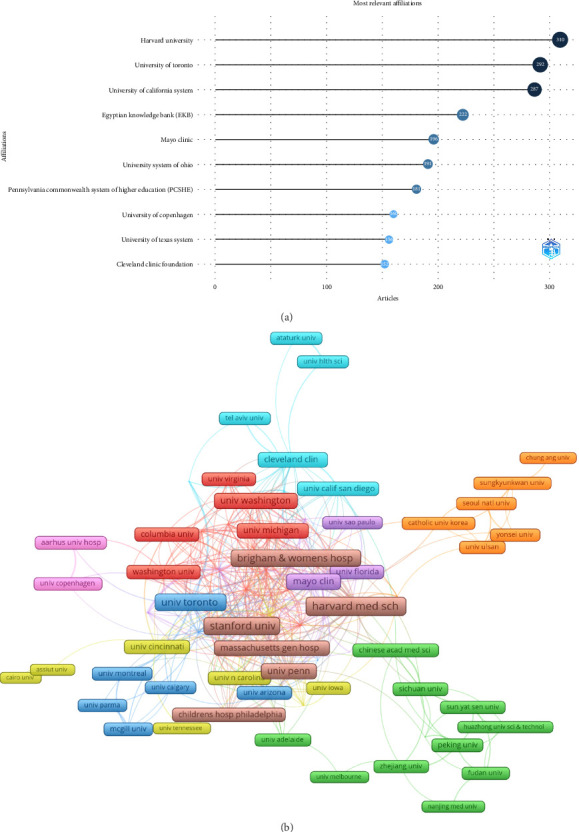
(a) Top 10 institutions by article count and rank. The circle size shows the article count, with darker shades indicating higher ranks. (b) Visualization map depicting the collaboration among different institutions. Nodes represent institutions, with size indicating publication count. Links represent co-authorships, with thickness showing collaboration strength. Colors indicate different research clusters. Total link strength in collaboration networks measures the frequency of co-authorship between institutions, indicating the level of collaborative research.

From an institutional perspective, Harvard University (310), the University of Toronto (292), and the University of California system (287) had the highest publication counts (Figure 4(a)). A collaboration network for 75 institutions with at least 20 publications was constructed (Figure 4(b)). Harvard Medical School had the highest number of collaborations with other countries (91), followed by Stanford University (81) and Duke University (64). The University of Toronto ranked fourth with 59 collaborations. Institutions with high publication counts also tended to rank high in collaboration strength, underscoring the exceptional research capacity and collaborative nature of USA institutions. In contrast, despite China's second overall publication rank, the Chinese Academy of Medical Sciences had a low total link strength of 16, indicating a need for more international collaboration in this field (Figure 4(b)).

### 3.4. Global Collaboration Network of Journals

The three most influential journals identified in this study are Anesthesia and Analgesia, with 9445 citations, an h-index of 31, a total publication count of 96, and a total publication rank of 5; Anesthesiology, which has 7485 citations, an h-index of 29, a total publication count of 57, and a total publication rank of 13; and the Journal of Arthroplasty, which reports 2267 citations, an h-index of 27, a total publication count of 101, and a total publication rank of 4 (Table 2). Their impact factors were 4.6, 9.1, and 3.4, respectively, with all three journals ranked in the first quartile (Q1) of the JCR. Notably, among the top 20 journals by h-index, Anesthesiology had the highest impact factor, highlighting its significant influence in the field.

**Figure 5:**
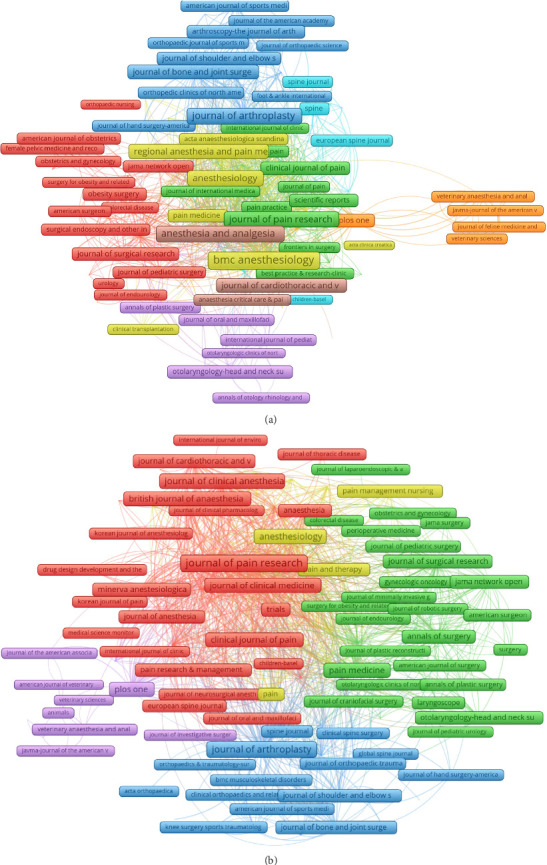
Visualization map depicting the co-occurrence networks of journals and coupling networks. (a) The co-occurrence networks of journals. The frequency with which journals are cited together within the same articles reflects thematic or topical connections between the research they publish. (b) The coupling networks of journals. The extent to which journals are linked is based on common references cited in their articles, indicating a shared intellectual foundation or research focus.

A total of 30 journals with at least 2 related publications were selected, and co-occurrence networks were created. The three key journals with the highest total link strength in co-occurrence networks were BMC Anesthesiology (582), Anesthesia and Analgesia (442), and Journal of Arthroplasty (387). This highlighted the significant research focus on opioids in the field of postoperative analgesia, emphasizing these journals' roles in shaping and disseminating influential studies within this area (Figure 5(a)). Coupling networks evaluated shared references among journals, where strong link strength signified significant reference overlap, indicating a common research foundation. The three key journals with the highest total link strength in co-occurrence networks were Journal of Pain Research (39,520), BMC Anesthesiology (38,026), and Anesthesia and Analgesia (29,172) (Figure 5(b)).

### 3.5. Global Collaboration Network of Authors

**Figure 6:**
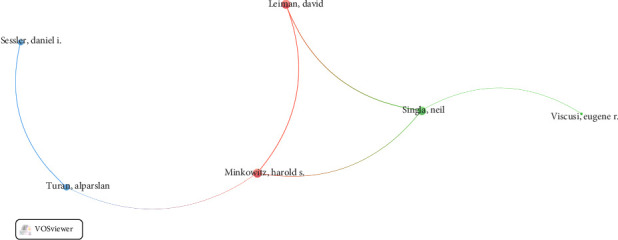
Visualization map depicting the collaboration among different authors. Nodes represent authors, with size indicating publication count. Nodes represent authors, with size indicating publication count. Links represent co-authorships, with thickness showing collaboration strength. Colors indicate different research clusters. Total link strength in collaboration networks measures the frequency of co-authorship between authors, indicating the level of collaborative research.

A total of 27,831 authors contributed to research on opioids in the field of postoperative analgesia. Among the top 10 authors, Meissner Winfried held the highest h-index of 12, reflecting a significant scholarly presence with 2 publications and a citation count of 520. Brummett Chad M., Kang Hyun and Mariano Edward R. exhibited a strong research profile and had h-index of 10 each, accompanied by 14, 1, and 11 publications and 646, 287, and 362 citations, respectively. Notably, citation count and h-index did not always correlate; for instance, Bateman Brian T. had the highest total citation count of 906 but an h-index of 9, while Kang Hyun, with only 287 citations, had an h-index of 10 (Table 3). A collaboration network was constructed for authors with at least 10 publications. Among the 35 authors engaged in international collaborations with 10 or more articles, Mathieson Ole led with 45 collaborations, followed by Hagi Pedersen Daniel (38) and Chae Min Suk (34) (Figure 6).

### 3.6. Analysis of High-Cited References

The top 10 most cited works in the field of opioid use for postoperative analgesia, ranked by total citations, are listed in Table 4. The citations in the top 10 ranged from 710 to 275. Among the top 10 papers by total citation frequency, Hill et al., published in Annals of Surgery in 2017, had the greatest overall citation frequency (710) [17], followed by the article published in Lancet in 2019 by Glare et al. (464) [18] and European Journal of Pain in 2018 by Arendt-Nielsen et al. (415) [19].

### 3.7. Analysis of Keyword Co-Occurrence and Burst Keyword

**Figure 7:**
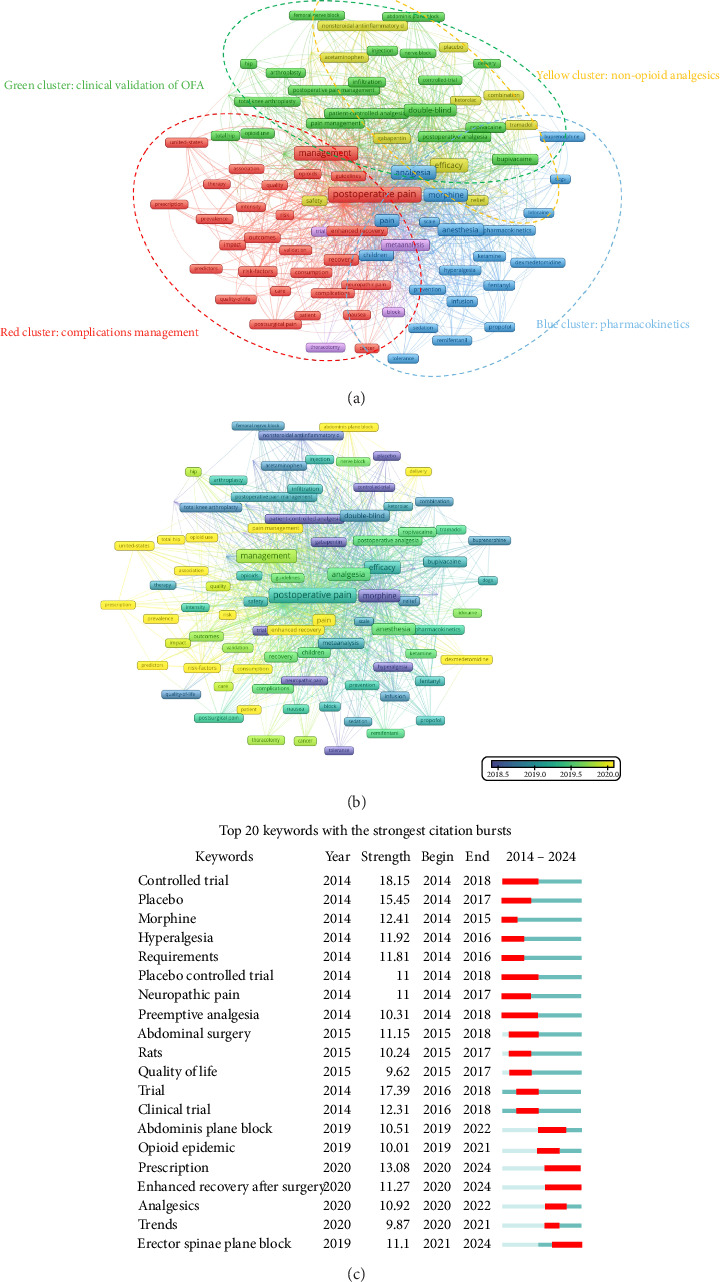
(a) Visual analysis of keyword co-occurrence network analysis. Each node represents a keyword, with size indicating its frequency of occurrence. Links between nodes represent co-occurrence in the same documents, with thicker lines showing stronger associations. Colors reflect the cluster of the articles. (b) Visual analysis of keyword co-occurrence network and timeline change analysis. Each node represents a keyword, with size indicating its frequency of occurrence. Links between nodes represent co-occurrence in the same documents, with thicker lines showing stronger associations. Colors reflect the average publication year of the articles, as indicated by the color gradient at the bottom right. The transition from purple to green to yellow represents the timeline of keywords, with purple indicating older terms and yellow representing the most recent ones. (c) Top 20 keywords with the strongest citation bursts (CiteSpace). Top 20 keywords with the strongest citation bursts from 2014 to 2024. The blue lines represent the period, and the red lines indicate the burst periods of the keywords.

A total of 134 keywords with at least 20 occurrences were identified, enabling the rapid identification of research hotspots in the field. As illustrated in Figure 7(a), the keywords were grouped into four clusters: the red cluster focused on complications management, emphasizing patient outcomes and postoperative management, including terms such as “outcomes,” “quality of life,” “management,” and “complication.” The green cluster emphasized clinical validation of OFA, highlighting the validation of safety and efficacy, featuring keywords like “double-blind” and “controlled-trial.” The yellow cluster covered nonopioid analgesics, including terms like “combination,” “efficacy,” and “gabapentin.” Finally, the blue cluster highlighted pharmacokinetics, requiring special attention in appropriate dosing regimens, such as “pharmacokinetics” and “prevention” (Figure 7(a)).

The development of research over the past decade was depicted, with keywords arranged by time (Figure 7(b)). The purple dots represented keywords at mid-2018, reflecting earlier research priorities focused on specific drugs, such as “morphine,” “gabapentin,” and “nonsteroidal anti-inflammatory.” This suggested that research at that time was in its early stages, concentrating on different types of medications. The green dots, which indicated mature research trends around 2019, highlighted an emphasis on keywords such as “efficacy,” “management,” “pharmacokinetics,” and “anesthesia,” showing that studies had become more in-depth, focusing on the efficacy and management of opioids, especially given their addictive potential, as well as pharmacokinetics and anesthesiology to improve postoperative analgesia. Finally, the yellow dots signified emerging trends at 2020, with a focus on terms like “risk factors,” “consumption,” and “prescription,” indicating a growing emphasis on safer pain management strategies to reduce opioid consumption (Figure 7(b)).

Keyword burst analysis revealed emerging terms, highlighting shifts in research focus and identifying areas of growing interest in the field. The earliest burst appeared in 2014, and the most recent in 2020. The strongest burst was observed for “controlled trial,” while the latest bursts included “prescription,” “enhanced recovery,” and “Erector Spinae Plane Block.” Overall, burst strengths ranged from 9.62 to 18.15, with durations of up to 5 years. These bursts showed a high degree of overlap with keyword co-occurrence patterns, indicating consistent research trends aligned with keyword co-occurrence (Figure 7(c)).

## 4. Discussion

This study primarily summarizes the key contributors to research on opioids in postoperative analgesia, highlighting the leading countries (mainly the USA), influential institutions (predominantly USA-based, such as Harvard University and the University of California system), and impactful journals like Anesthesia and Analgesia. Prominent authors, including Meissner Winfried, were also identified. Importantly, we have traced the development trends in opioid research for postoperative pain management. The focus has evolved from early studies on single drugs, such as morphine, to more in-depth investigations emphasizing pharmacokinetics. More recently, research has shifted towards the implementation and widespread adoption of enhanced recovery protocols, reflecting a more patient-centered and scientifically driven approach to opioid use in postoperative settings. Consequently, our bibliometric analysis provides valuable insights into the research landscape and identifies emerging hotspots for future investigations.

### 4.1. Influence of the Top 3 Most Cited Articles

In the first high-cited article, they found that current opioid prescribing practices for common surgical procedures demonstrate considerable inconsistency, with many patients receiving more medication than clinically necessary. This may result in rising mortality from opioid [17], highlighting rational dose of dosage of opioid prescriptions to decrease the amount of unused opioid pills available for misuse, abuse, or diversion.

The second high-cited article discussed advances in the prevention of transition from acute postoperative pain to persistent pain that are unresponsive to opioids due to opioid-induced hyperalgesia [18]. To eliminate the overprescribing of opioids after surgery, a comprehensive biopsychosocial approach is necessary to treatment postoperative chronic pain. Consequently, transitional pain service represents an innovative model designed to address this situation [20].

The third high-cited article explored the assessment and manifestation of central sensitization, which may be a potential new option to develop therapies and profile drugs for chronic postoperative pain [19]. Preclinical studies confirm opioids modulate descending inhibitory pathways to modulate the interaction between central sensitization and chronic pain [21]. Identification of receptor and molecular targets involved in this process facilitate the development of novel opioid and opioid-free analgesic therapies in the future [22].

### 4.2. Research Hotspots

The keyword co-occurrence analysis identified four main clusters, focusing on complications management, validation of safety and efficacy, combination anesthesia plan, and pharmacokinetics.

#### 4.2.1. Cluster 1 (Red): Complications Management

This cluster included the keywords like “outcomes,” “quality of life,” “management,” and “complications.” The central nervous system (CNS) is central to opioid overdose because it not only controls breathing but is also highly susceptible to oxygen deficiency resulting from respiratory depression, which is responsible for opioid-induced fatalities as well as the short- and long-term morbidities linked to a nonfatal overdose [23]. Hypoxic brain injury caused by opioid overdose can also lead to various neurological complications, such as seizures, transient paralysis, coma, and stroke. Other consequences may include confusion, memory impairment, loss of coordination, walking difficulties, paraplegia, catatonia, slowed reflexes, and impaired motor function [24]. Although high-affinity, traditional extended-duration opioid antagonists (e.g., Naloxone) have therapeutic value in overdose reversal, they may provoke significant and protracted withdrawal in opioid-dependent patients, including agitation, piloerection, nausea and vomiting, diarrhea, sweating, muscle cramps, hypertension, and tachycardia. Furthermore, scholars have noted that “overantagonism” poses risks of iatrogenic complications, such as pulmonary edema and angina [25]. Thus, advancement of more potent overdose reversal agents should be considered a public health imperative, as it plays a vital role in minimizing the postoperative complications by opioid misuse [26]. Besides, encouragement of nonopioid therapies for perioperative anesthesia by combination therapy should be highlighted to curtail unnecessary prescription and reduce opioid consumption [27].

#### 4.2.2. Cluster 2 (Yellow): Nonopioid Analgesics

This cluster included the keywords like “nonsteroidal antiinflammation,” “efficacy,” and “gabapentin.” OFA is defined by the complete avoidance of opioids during anesthesia, instead utilizing integrated approaches that may include nerve blocks along with nonopioid analgesics [28]. By minimizing or eliminating opioid use, these techniques may improve surgical recovery parameters and prevent narcotic-related side effects [29]. The commonly used nonopioid analgesics include ketamine, dexmedetomidine, lidocaine, and nonsteroidal anti-inflammatory drugs [28]. A recent network meta-analysis showed that lidocaine (strongest effect), ketamine, and gabapentinoids may attenuate chronic postsurgical pain within 6 months, though the certainty of evidence remains limited [30].

#### 4.2.3. Cluster 3 (Green): Clinical Validation of OFA

This cluster included the keywords like “double-blind” and “controlled-trial.” Recent studies focus on optimizing intervention techniques, such as nerve blocks along with nonopioid analgesics. Published double-blind randomized controlled trials continue to validate the effectiveness of various analgesic strategies and highlight clinical trials of novel administration methods, such as local anesthesia and ultrasound-guided nerve blocks, demonstrating their potential to reduce the need for opioid use postoperatively [31, 32]. Current evidence from previous four meta-analyses comparing OFA and opioid-based anesthesia indicates that OFA significantly reduces postoperative nausea/vomiting (PONV) and recovery-period complications. However, no significant differences were observed in postoperative pain control or opioid requirements [33–36]. It is suggested that with refinement of OFA techniques, further research in the future may reveal more divergence between OFA and opioid-based anesthesia, in particular for those more at risk of opioid-related side effects and/or challenging postoperative pain and complications.

#### 4.2.4. Cluster 4 (Blue): Pharmacokinetics

This cluster included the keywords like “pharmacokinetics” and “prevention.” The abuse liability of opioid compounds is fundamentally determined by their pharmacological characteristics, particularly pharmacokinetic properties that influence drug concentration and activity at CNS receptor sites [37]. The physicochemical properties of the drug, route of administration, rate of administration, and onset effect rate are key pharmacokinetics properties that influence abuse potential. Fentanyl, oxycodone, and the di-acetylated morphine prodrug heroin are known to be highly lipophilic and rapidly absorbed across blood–brain barrier [38]. The abuse liability of opioids varies significantly with administration method, where formulations with efficient absorption via oral or nasal mucosa achieve more rapid and complete systemic distribution, thereby increasing their misuse potential [39]. Besides, the risk of fatal opioid overdose increases proportionally with dosage. Extended-release opioid formulations, such as hydromorphone and oxycodone, mitigate this risk through dose-limiting pharmacokinetics via longer time to maximum concentration [40]. Additionally, rapid drug absorption kinetics may potentially lead to increased systemic exposure and enhanced pharmacological effects. Taken together, further pharmacokinetic-pharmacodynamic investigations are required to elucidate the mechanistic relationship between drug absorption characteristics and abuse liability, which can inform the development of next-generation opioids with improved safety profiles and enhanced abuse-deterrent properties.

### 4.3. Research Frontiers

In recent years, the “Enhanced Recovery After Surgery (ERAS)” approach has become increasingly important in postoperative care [41]. ERAS aims to minimize complications, shorten hospital stays, and accelerate recovery through multidisciplinary collaboration and optimized perioperative protocols. Effective pain management is a key factor within this framework, and the judicious use of opioids directly impacts patient outcomes [41]. Over-reliance on opioids can result in complications like nausea, vomiting, respiratory depression, and severe opioid-related adverse events, hindering recovery. Thus, ERAS advocates for multimodal analgesia strategies to minimize opioid use and associated side effects [42]. These strategies include using NSAIDs, acetaminophen, and local anesthetic techniques in conjunction. Studies have shown that multimodal analgesia effectively controls postoperative pain while reducing opioid requirements and adverse reactions. For example, a 2024 study demonstrated that implementing multimodal analgesia within the ERAS pathway significantly reduced opioid consumption and improved recovery quality [43]. Additionally, ERAS emphasizes preoperative patient education and expectation management, encouraging active participation in the recovery process, which helps decrease opioid dependence and promote early mobilization and functional recovery [44].

One promising technique for scientifically reducing opioid use is the “Erector Spinae Plane Block (ESPB),” an emerging regional nerve block [45]. By injecting local anesthetics between the erector spinae muscle and the transverse process, ESPB blocks multiple spinal nerves, providing effective postoperative pain relief. Proven effective for thoracic, abdominal, and back surgeries, ESPB is simple to perform, safe, and associated with minimal side effects [46]. Recent studies have shown that incorporating ESPB into pain management protocols can significantly reduce opioid requirements and their associated adverse effects. For instance, a 2021 study found that ESPB reduced opioid consumption within the first 24 h after thoracic surgery, improving patient comfort and overall recovery [47]. Integrating ESPB into multimodal analgesia strategies offers sustained pain relief, aligning with advancements in ERAS.

### 4.4. Strengths and Limitations

The strengths of this study include its comprehensive bibliometric analysis of the literature on opioids in postoperative analgesia, providing valuable insights into research trends, influential authors, and key publications. The use of multiple databases and analysis tools enhances the reliability of the findings, while the focus on recent developments highlights the evolving research landscape in this field. However, limitations exist, such as potential publication bias, since only English-language articles were included, which may have excluded relevant studies published in other languages. Additionally, the analysis is based on available publications, so the conclusions may not fully capture ongoing research efforts or emerging trends beyond the analyzed publication period.

## 5. Conclusion

This bibliometric analysis mapped the scientific landscape of opioids in postoperative analgesia, highlighting key contributions, research trends, and potential future directions. The study offered a quantitative assessment of the field, aimed at assisting researchers, clinicians, and policymakers in understanding the evolving dynamics of pain management and the role of opioids based on global collaboration network of authors, journals, and institutions. The findings emphasized the importance of complications management, nonopioid analgesics, clinical validation of OFA, and pharmacokinetics of opioids as research hotspots. These findings underscore the growing interest in improving patient outcomes through individual-based multimodal analgesia to deepen insights into pain mechanisms and inform the development of more effective and safer postoperative pain management strategies based on global collaboration network.

## Figures and Tables

**Table 1:** Publication and citation profiles of leading countries.

Country	Articles	Freq	SCP	MCP	MCP_ratio	TP	TP_rank	TC
USA	1884	35	169	1715	0.090	6608	2	31,954
China	768	14.3	49	719	0.064	2089	3	7062
South Korea	308	5.7	19	289	0.062	812	5	3606
Turkey	206	3.8	9	197	0.044	549	7	2970
Canada	189	3.5	54	135	0.286	867	4	4053
France	135	2.5	22	113	0.163	637	6	2361
Italy	129	2.4	29	100	0.225	512	8	1869
Brazil	126	2.3	18	108	0.143	377	13	1361
Germany	124	2.3	34	90	0.274	451	9	1516
Egypt	118	2.2	7	111	0.059	225	19	1280
The United Kingdom	103	1.9	41	62	0.398	399	12	1856
Denmark	97	1.8	17	80	0.175	418	10	2444
Japan	94	1.7	5	89	0.053	248	15	1031
Australia	90	1.7	23	67	0.256	399	11	1762
India	90	1.7	7	83	0.078	225	20	1548
Iran	77	1.4	5	72	0.065	238	18	685
The Netherlands	70	1.3	15	55	0.214	335	14	1443
Poland	60	1.1	8	52	0.133	241	17	542
Thailand	59	1.1	11	48	0.186	147	25	404
Spain	56	1	6	50	0.107	243	16	615

*Note:* Articles: Publications of Corresponding Authors only. Freq: Frequence of Total Publications. MCP_Ratio: Proportion of Multiple Country Publications. TP_rank: Rank of Total Publications.

Abbreviations: TC = total citations, TP = total publications.

**Table 2:** Bibliometric indicators of high-impact journals.

Journal	h_index	g-index	m-index	TP	TP_rank	TC	TC_rank	PY_start	IF_2023	JCR_2023
Anesthesia and Analgesia	31	46	2.818	96	5	9445	1	2014	4.6	1
Anesthesiology	29	48	2.636	57	13	7485	2	2014	9.1	1
Journal of Arthroplasty	27	44	2.455	101	4	2267	7	2014	3.4	1
Regional Anesthesia and Pain Medicine	25	43	2.273	64	10	3549	5	2014	5.1	1
British Journal of Anesthesia	24	36	2.182	54	16	6637	3	2014	9.1	1
Journal of Clinical Anesthesia	24	38	2.182	96	6	2129	9	2014	5.0	1
Plos One	24	40	2.182	76	8	1177	23	2014	2.9	1
BMC Anesthesiology	23	34	2.091	160	1	784	34	2014	2.3	2
Journal of Cardiothoracic and Vascular Anesthesia	21	39	2.100	75	9	999	25	2015	2.3	2
Journal of Pain Research	21	32	1.909	151	2	1269	19	2014	2.5	2
Pain	21	36	1.909	36	27	5080	4	2014	5.9	1
Journal of Anesthesia	19	27	1.727	53	19	677	38	2014	2.8	2
Medicine	19	25	1.900	110	3	836	30	2015	1.3	2
Anesthesia	18	36	1.636	37	26	2480	6	2014	7.5	1
European Journal of Anaesthesiology	18	32	1.636	53	18	1546	12	2014	4.2	1
Annals of Surgery	17	25	2.125	25	45	1467	14	2017	7.5	1
Clinical Journal of Pain	17	23	1.545	57	14	1405	16	2014	2.6	2
Pain Medicine	17	25	1.545	50	20	1305	18	2014	2.9	1
Pediatric Anesthesia	17	27	1.545	61	11	1466	15	2014	1.7	2
Journal of Bone and Joint Surgery-American Volume	16	28	1.600	28	37	1606	11	2015	4.4	1

*Note:* h_index: The h-index of the journal, which measures both the productivity and citation impact of the publications. g_index: The g-index of the journal, which gives more weight to highly-cited articles. m_index: The m-index of the journal, which is the h-index divided by the number of years since the first published paper. IF_2023: Impact factor in 2023, indicating the average number of citations to recent articles published in the journal. JCR_2023: The quartile ranking of the journal in the Journal Citation Reports in 2023, indicating the journal's ranking relative to others in the same field (Q1: top 25%, Q2: 25%–50%, Q3: 50%–75%, Q4: bottom 25%). TP_rank: Rank of Total Publications. TC_rank: Rank of Total Citations. Average Citations: The average number of citations per publication. PY_start: Publication Year Start, indicating the year the journal started publication.

Abbreviations: TC = total citations, TP = total publications.

**Table 3:** Publication and citation profiles of high-impact authors.

Authors	h_index	g-index	m-index	PY_start	TP	TP_frac	TP_rank	TC	TC_rank
Meissner Winfried	12	22	1.09	2014	23	3.43	1	520	4
Brummett Chad M.	10	14	1.00	2015	14	1.74	9	646	2
Kang Hyun	10	16	0.91	2014	18	3.40	2	287	11
Mariano Edward R.	10	11	0.91	2014	11	1.13	24	362	8
Ahiskalioglu Ali	9	13	0.90	2015	13	1.93	10	372	7
Bateman Brian T.	9	14	1.00	2016	14	2.31	8	906	1
Clarke Hance	9	12	0.82	2014	12	1.62	15	572	3
Habib Ashraf S.	9	11	0.82	2014	11	2.36	22	221	19
Minkowitz Harold S.	9	12	0.82	2014	12	2.31	17	377	6
Mont Michael A.	9	9	0.82	2014	9	1.31	46	496	5
Sessler Daniel I.	9	12	0.82	2014	12	1.21	18	287	11
Turan Alparslan	9	15	0.82	2014	15	1.84	5	245	17
Wang Qiuru	9	14	1.50	2019	15	2.65	7	218	20
Allegri Massimo	8	9	0.80	2015	9	0.70	38	198	22
Bugada Dario	8	11	0.80	2015	11	1.39	20	245	17
Clark J. David	8	9	0.73	2014	9	1.52	41	289	10
Kang Pengde	8	13	1.33	2019	13	2.29	11	188	24
Katz Joel	8	12	0.80	2015	12	1.38	16	210	21
Mathiesen Ole	8	15	0.73	2014	15	1.50	4	283	13
Okoroha Kelechi R.	8	9	0.89	2016	9	1.39	47	271	15

*Note:* h_index: The h-index of the journal, which measures both the productivity and citation impact of the publications. g_index: The g-index of the journal, which gives more weight to highly-cited articles. m_index: The m-index of the journal, which is the h-index divided by the number of years since the first published paper. TP_rank: Rank of Total Publications. TC_rank: Rank of Total Citations. PY_start: Publication Year Start, indicating the year the journal started publication. TC_rank: Rank of Total Citations. PY_start: Publication Year Start, indicating the year the journal started publication.

Abbreviations: TC = total citations, TP = total publications.

**Table 4:** Top 10 high-cited references.

Paper	Doi	Title	TC	Journal
Hill M. V., 2017, Annals of Surgery	10.1097/SLA.0000000000001993	Wide variation and excessive dosage of opioid prescriptions for common general surgical procedures	710	Annals of Surgery
Glare P., 2019, Lancet	10.1016/S0140-6736(19)30352-6	Transition from acute to chronic pain after surgery	464	Lancet
Arendt-Nielsen L., 2018, European Journal of Pain	10.1002/ejp.1140	Assessment and manifestation of central sensitization across different chronic pain conditions	415	European journal of Pain
Clarke H., 2014, BMJ: British Medical Journal	10.1136/bmj.g1251	Rates and risk factors for prolonged opioid use after major surgery: Population-based cohort study	393	BMJ
Dalla Costa E., 2014, Plos One	10.1371/journal.pone.0092281	Development of the horse grimace scale (HGS) as a pain assessment tool in horses undergoing routine castration	356	PLoS ONE
Thiele R. H., 2015, Journal of the American College of Surgeons	10.1016/j.jamcollsurg.2014.12.042	Standardization of care: impact of an enhanced recovery protocol on length of stay, complications, and direct costs after colorectal surgery	308	Journal of the American College of Surgeons
Hill M. V., 2018, Annals of Surgery	10.1097/SLA.0000000000002198	An educational intervention decreases opioid prescribing after general surgical operations	294	Annals of surgery
Chin K. J,, 2017, Anaesthesia	10.1111/anae.13814	The analgesic efficacy of preoperative bilateral erector spinae plane (ESP) blocks in patients having ventral hernia repair	292	Anaesthesia
Neuman M. D., 2019, Lancet	10.1016/S0140-6736(19)30428-3	Inappropriate opioid prescription after surgery	284	Lancet
Schwenk E. S., 2018, Regional Anesthesia & Pain Medicine	10.1097/AAP.0000000000000806	Consensus guidelines on the use of intravenous ketamine infusions for acute pain management from the American society of regional anesthesia and pain medicine, the American Academy of pain medicine, and the American society of Anesthesiologists	275	Regional Anesthesia & Pain Medicine

Abbreviation: TC = total citations.

## Data Availability

All data generated or analyzed during this study are included in this published article.
